# Tunable Plasmon Resonance in Silver Nanodisk-on-Mirror Structures and Scattering Enhancement by Annealing

**DOI:** 10.3390/nano14191559

**Published:** 2024-09-26

**Authors:** Ryohei Hatsuoka, Kota Yamasaki, Kenji Wada, Tetsuya Matsuyama, Koichi Okamoto

**Affiliations:** 1 Department of Physics and Electronics, Graduate School of Engineering, Osaka Metropolitan University, 1-1 Gakuen-cho, Naka-ku, Sakai 599-8531, Osaka, Japan; 2 Equipment Sharing Center for Advanced Research and Innovation, Osaka Metropolitan University, 1-1 Gakuen-cho, Naka-ku, Sakai 599-8531, Osaka, Japan; wada.kenji@omu.ac.jp

**Keywords:** plasmonics, metamaterials, localized surface plasmon resonance

## Abstract

In this study, we evaluated the surface plasmon characteristics of periodic silver nanodisk structures fabricated on a dielectric thin-film spacer layer on a Ag mirror substrate (NanoDisk on Mirror: NDoM) through finite difference time domain (FDTD) simulations and experiments involving actual sample fabrication. Through FDTD simulations, it was confirmed that the NDoM structure exhibits two sharp peaks in the visible range, and by adjusting the thickness of the spacer layer and the size of the nanodisk structure, sharp peaks can be obtained across the entire visible range. Additionally, we fabricated the NDoM structure using electron beam lithography (EBL) and experimentally confirmed that the obtained peaks matched the simulation results. Furthermore, we discovered that applying annealing at an appropriate temperature to the fabricated structure enables the adjustment of the resonance peak wavelength and enhances the scattering intensity by approximately five times. This enhancement is believed to result from changes in the shape and size of the nanodisk structure, as well as a reduction in grain boundaries in the metal crystal due to annealing. These results have the potential to contribute to technological advancements in various application fields, such as optical sensing and emission enhancement.

## 1. Introduction

The phenomenon where metal structures are miniaturized to a nanoscale smaller than the wavelength, thereby confining the electric field within the nanostructure and causing strong scattering and absorption at specific wavelengths, is called localized surface plasmon resonance (LSPR) [1]. The resonance conditions and effects of LSPR vary depending on the shape, size, and material of the nanostructure, as well as the properties of the surrounding medium. Typically, Au or Ag nanoparticles are used to achieve resonance in the visible light range.

In recent years, the plasmonic colors of various metal nanostructures have attracted attention as applications of LSPR [2,3,4,5,6]. Advances in nanofabrication techniques have enabled the manufacture of nanoscale metal structures, allowing for the creation of nanostructures of various sizes and shapes. As a result, many research groups have reported plasmonic colors using metal nanostructures, including nanodisk arrays [7,8,9,10,11,12], nanohole arrays [8,13,14,15,16], nanoantennas [17,18,19], nanorods [3,20,21,22,23], apertures [2,13], and gratings [24,25,26,27,28,29,30,31,32,33,34,35].

Among metal nanostructures with LSPR, the electromagnetic field response of Ag microspheres in air is characterized by a single, broad absorption peak. This broad peak means that the wavelengths of absorbed light are not limited, resulting in only pastel-like, low-saturation colors. However, in our previous study, we discovered that by depositing a thick Ag film on a glass substrate, then depositing an Al_2_O_3_ spacer layer on top of it, and subsequently depositing a thin Ag film on the spacer layer, followed by annealing to form Ag nano-hemisphere structures, two sharp resonance peaks appear in the visible range. This structure, named the Nano-Hemisphere on Mirror (NHoM) structure [36,37], has been reported for its application as a colorimetric sensor [38]. The presence of two peaks in the visible range provides the advantages of representing color mixing and achieving high-sensitivity sensing, as each resonance peak responds to changes in the surrounding environment. Additionally, if the resonance peak wavelength can be controlled with high precision, it becomes possible to selectively adjust the resonance wavelength range according to the application.

In the NHoM structure, nano-hemisphere structures are formed by annealing thinly deposited metal, resulting in random arrangements and variations in particle size. Despite this random fabrication method, sharp peaks have been observed. Further sharpening of the peaks can be expected by arranging the nanostructures in an orderly manner and eliminating randomness. Therefore, in this study, we aimed to eliminate the randomness of the previous NHoM structure by using microfabrication techniques to create a periodic and uniform silver nanodisk structure on a mirror substrate, achieving sharper resonance peaks in the visible range and more precise control of the peak wavelengths. Ag was used to achieve a sharp plasmon resonance in the visible region, as it is known to exhibit sharper plasmon resonance peaks compared to other metals, such as Au and Al.

In addition, we have reported on the optical properties of periodic metal nanodisk structures fabricated on a glass substrate (NanoDisk on Glass: NDoG) using EBL [39]. We found that annealing the fabricated structures improved their plasmonic properties. Based on this finding, we applied similar annealing treatments to the nanostructures on the mirror substrate fabricated in this study and investigated changes in their optical properties, including control of the resonance peak wavelength.

## 2. Materials and Methods

### 2.1. Calculations

In this study, we performed the modeling of the nanostructures and the calculation of their electric field responses using the FDTD method. Commercial software (Poynting for Optics V3L10, Fujitsu, Tokyo, Japan) was used for these FDTD simulations. In the computational model, the surrounding refractive index was set to 1, and the Ag mirror layer was made sufficiently thick (at least 50 nm) to exceed the penetration depth of the surface plasmon resonance. This ensured that the plasmon effect is confined to the vicinity of the particle surface. A dielectric spacer layer was placed on top of the Ag mirror layer, and a Ag nanodisk was placed on top of the spacer layer. The dielectric constant of Ag was approximated using the Drude model based on the reported values by Johnson and Christy [40]. In this computational model, absorbing boundary conditions were applied in the z-axis direction, and periodic boundary conditions were set in the x- and y-axis directions. The period in the x and y directions was fixed at 200 nm. A differential Gaussian model pulse light with a pulse width of 0.3 fs and an electric field intensity of 1 V/m was used as the light source. The peak position of the excitation pulse was approximately 600 THz (wavelength 500 nm). The measurement time was adjusted within the range of 10 to 40 fs for each model. The reflectance obtained from the simulation was converted into an extinction spectrum using the following equation:

$$Extinction=-{log}_{10}\left(\frac{R}{R_{0}}\right)$$

where *R* and *R*_0_ are the reflectance of the NDoM structure and the mirror substrate (without the nanodisk), respectively. The scattering intensity was calculated as the sum of the electric field intensity ${\left|E\right|}^{2}$ of each angular component.

### 2.2. Fabrication Method

**Figure 1:**
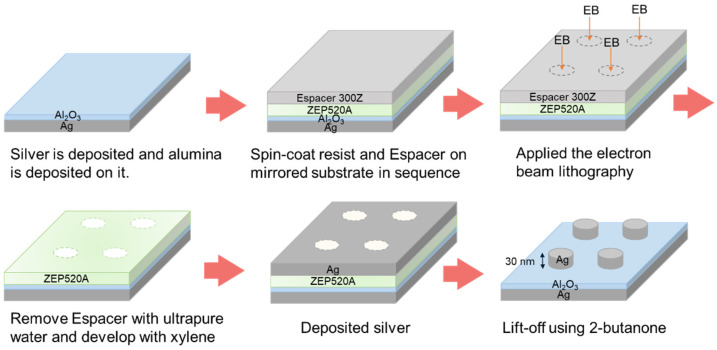
Fabrication procedure of NDoM structure.

Figure 1 shows the fabrication process of the NDoM structure. First, a mirror substrate was fabricated by depositing 50 nm of Ag followed by 15 nm of Al_2_O_3_ on a cover glass (No. 3, Matsunami Glass Industry, Osaka, Japan) using a multi-target RF sputter (FRS-2CP-260T, FKD Factory, Tokyo, Japan). On the fabricated mirror substrate, ZEP-520A (Zeon Corporation, Tokyo, Japan) was spin-coated as the resist, followed by Espacer 300Z (Showa Denko K.K., Tokyo, Japan) as the anti-charging agent. The spin-coating of ZEP-520A was performed at 500 rpm for 20 s, followed by a 5 s slope, then at 5000 rpm for 120 s, and subsequently heated at 180 °C for 180 s. The spin-coating of Espacer 300Z was performed at 300 rpm for 30 s, followed by a 5 s slope, then 1500 rpm for 60 s, and subsequently heated at 100 °C for 60 s. Next, using an EBL system (ELS-7500EX, Elionix, Tokyo, Japan), the nanodisk structures were patterned under the conditions of a beam current of 100 pA and a dose time of 1.2 µs. The nanodisk structures, with a radius of 40 nm and an inter-disk distance of 120 nm, were modeled in a 300 × 300 µm area using AutoCAD (Autodesk, San Rafael, CA, USA). After patterning, the samples were immersed in ultrapure water for 1 min to remove the Espacer, followed by development in xylene for 1 min. Subsequently, 30 nm of Ag was deposited using a resistive heating vacuum deposition system (SVC-700TM, Sanyu Electron, Tokyo, Japan) at a chamber pressure of 1.1 × 10^−3^ Pa and a deposition rate of 2.0 to 3.0 Å/s. Finally, lift-off was performed using 2-butanone to create the Ag nanodisk pattern structure. To further enhance the optical properties of the fabricated structures, annealing was performed in an electric furnace (FO100, Yamato Scientific Co., Ltd., Tokyo, Japan) under a nitrogen atmosphere for 5 min at each annealing temperature.

### 2.3. Observations and Measurements

The shape of the fabricated structures was observed using a thermionic emission scanning electron microscope (SEM) (FlexSEM 1000 II, Hitachi High-Tech, Tokyo, Japan). Additionally, the extinction spectrum was obtained by measuring the reflectance intensity of bright-field images observed with a hyperspectral camera (NH-OK2, EBA JAPAN, Tokyo, Japan) and converting it to the extinction spectrum. Furthermore, the scattering intensity was measured using dark-field images observed with a hyperspectral camera.

## 3. Results and Discussion

### 3.1. Calculations

**Figure 2:**
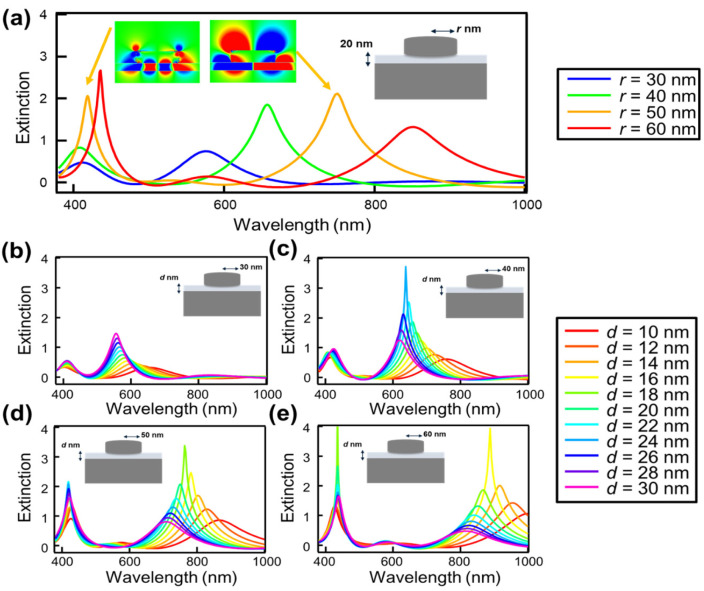
Dependence of the reflected extinction spectra of the NDoM structure calculated by FDTD simulation on (**a**) disk radius and thickness of the spacer layer for different radii: (**b**) *r* = 30 nm, (**c**) *r* = 40 nm, (**d**) *r* = 50 nm, and (**e**) *r* = 60 nm. (**a**) also shows the electric field distribution at the resonance peak wavelength for the nanodisk with a radius of 50 nm.

Figure 2 shows the peak wavelength and peak intensity of the localized surface plasmon resonance (LSPR) of the NDoM structure, as calculated using electromagnetic field analysis simulations via the FDTD method.

Figure 2a shows the reflection extinction spectra of the NDoM structure with the disk height fixed at 30 nm, the spacer layer thickness fixed at 20 nm, and the disk radius (*r*) varying from 30 nm to 60 nm in 10 nm increments. The results reveal that the reflection extinction spectra of the NDoM structure exhibit two resonance peaks in the visible range. This behavior is similar to that of the NHoM structure observed in previous research and arises due to the mirror image effect of the mirror substrate. The mirror image effect is a phenomenon where charges of opposite signs are induced within a metal to maintain a zero net charge at the metal’s surface when charges are present near the metal. The short-wavelength resonance peak corresponds to the quadrupole oscillation mode coupled with the mode induced in the mirror, while the long-wavelength resonance peak corresponds to the dipole oscillation mode coupled with the mode induced in the mirror [36,37]. The electric field distribution at the resonance peak wavelength for the nanodisk with a radius of 50 nm is shown in the inset of Figure 2a. From this distribution, it is evident that the short-wavelength resonance peak arises from the quadrupole oscillation mode, while the long-wavelength resonance peak results from the dipole oscillation mode, both coupled with the mode induced in the mirror.

It is known that in periodic metallic nanostructures, a strong resonance peak called surface lattice resonance (SLR) is observed due to the interaction between diffraction from the periodic structure and the evanescent field of plasmons [41,42,43]. However, since the period of the disk array is fixed at 200 nm, the peak wavelength of the SLR is 300 nm according to calculations based on the refractive index of the substrate, and since the upper side of the nanodisk structure is air, the resonance peak wavelength is calculated to be 200 nm. Therefore, the two observed resonance peaks are not considered to be due to the SLR effects but due to the interaction between the mirror substrate and the nanodisk.

When metallic nanostructures are periodically arranged and the momentum matching condition is satisfied, LSPR couples with propagating surface plasmon polaritons (SPPs). In such structures, the strong coupling between LSPR and SPPs results in sharper resonance peaks [44]. In the present study, however, periodic Ag nanostructures are placed on a thin dielectric layer of Al_2_O_3_, approximately 15 nm thick. Due to the characteristics of this structure, it is unlikely that a normal propagating SPP mode will be generated. Furthermore, since no excitation of propagating SPPs was observed in the simulations, it is concluded that the observed resonance peaks are mainly due to the interaction between the LSPR and the mirror substrate, rather than the coupling between LSPR and SPPs.

Additionally, as the disk radius increases, the resonance peaks shift towards longer wavelengths. This shift is attributed to the retardation effect, where an increase in particle size relative to the incident light wavelength causes a phase difference in the electric field of light on the incident and exit sides of the particles, leading to a delay in the collective oscillation of electrons.

Figure 2b–e show the dependence of the reflection extinction spectra of the NDoM structure on the thickness of the spacer layer (*d*), with the disk height fixed at 30 nm and radii set to 30 nm, 40 nm, 50 nm, and 60 nm, respectively. The spacer layer thickness was adjusted from 10 nm to 30 nm in increments of 2 nm. It is observed that adjusting the spacer layer thickness controls both the peak wavelength and intensity. Additionally, for all disk radii, the resonance peaks shift to longer wavelengths as the spacer layer thickness decreases. This shift is attributed to the reduced distance between the disk structure and the mirror substrate, which enhances the mode coupling.

**Figure 3:**
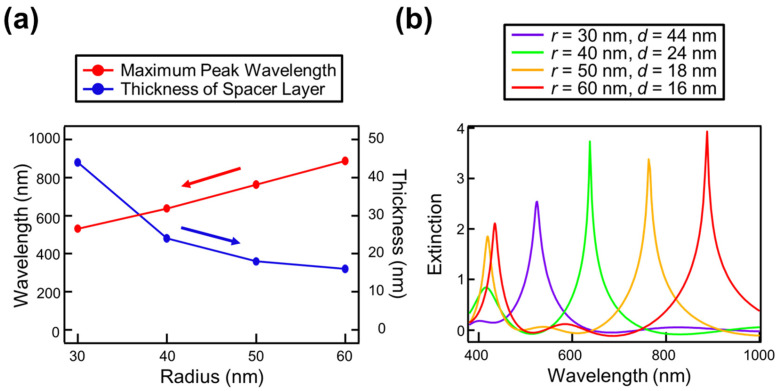
(**a**) The wavelength and spacer layer thickness for the maximum resonance peak intensity at each disk radius, and (**b**) the reflection extinction spectra when the resonance peak intensity is at its maximum.

Furthermore, it was found that the thickness of the spacer layer resulting in the maximum resonance peak intensity varies for each disk radius. Figure 3a shows the spacer layer thickness and the corresponding resonance peak wavelength for each disk radius at which the maximum peak intensity is achieved. Additionally, Figure 3b shows the reflection extinction spectra with the maximum intensity obtained for each disk radius.

From these results, it can be observed that as the disk radius increases, the thickness of the spacer layer required to achieve maximum peak intensity decreases. This is attributed to the fact that with a smaller disk radius, the electric field is more strongly localized at the disk’s sides. To adequately confine the electric field generated by the mirror image effect of the mirror substrate for such localized fields, a thicker spacer layer is necessary. The influence of the spacer layer is discussed in greater detail in our previous paper on the NHoM structure [36,45]. These findings indicate that sharp peaks can be obtained over a wide wavelength range by decreasing the thickness of the spacer layer as the disk diameter is increased. This suggests the possibility of achieving a full-color, high-performance colorimetric sensor using the NDoM structure.

### 3.2. Experiments

**Figure 4:**
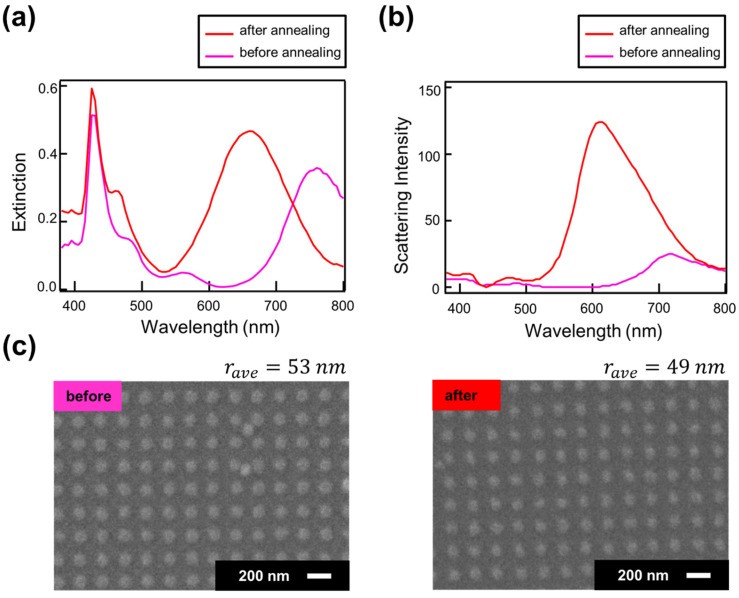
(**a**) Reflection extinction coefficient, (**b**) scattering intensity, and (**c**) SEM image of the NDoM structure before and after annealing.

Next, we fabricated the NDoM structure and experimentally investigated its plasmon resonance characteristics. A periodic silver disk structure with a height of 30 nm was fabricated on a mirror substrate composed of 50 nm of Ag and 15 nm of Al_2_O_3_ deposited on a glass substrate. The reflection extinction spectra and scattering spectra measured using a hyperspectral camera are shown in Figure 4a and Figure 4b, respectively. The surface morphologies, observed using SEM, are shown in Figure 4c. Additionally, the results after annealing at 150 °C for 5 min in a nitrogen atmosphere are also presented in Figure 4.

As shown in the reflection extinction spectrum in Figure 4a, the NDoM structure exhibits two resonance peaks in the visible range, similar to the numerical calculations. These peaks correspond to a short-wavelength peak due to the quadrupole oscillation mode coupled with the mode induced in the mirror, and a long-wavelength peak due to the dipole oscillation mode coupled with the mode induced in the mirror. Additionally, it is observed that the peak wavelength of the dipole mode shifts to shorter wavelengths after annealing. This shift is attributed to changes in the size of the disk structure caused by the annealing, as seen in Figure 4c. The observed shift of the resonance peak to shorter wavelengths due to the reduction in the structure size is consistent with the computational results shown in Figure 2. Furthermore, this finding suggests that in the NDoM structure, the resonance peak wavelength can be controlled not only by adjusting the disk size in electron beam lithography but also by varying the annealing conditions.

While the reflection extinction spectrum exhibits two resonance peaks, the scattering spectrum shown in Figure 4b exhibits only the peak of the dipole mode. This discrepancy is believed to arise because the coupled mode of the quadrupole mode and the mirror image mode becomes a dark mode, which is less likely to couple with the radiation field, resulting in a reduced scattering intensity.

Another notable point in Figure 4a,b is that, despite the reflection extinction intensity at the dipole mode resonance peak increasing by only about 1.2 times before and after annealing, the scattering intensity increases significantly by approximately 5 times. Since the simulation, which replicated the structural changes before and after annealing for the NDoM structure, did not confirm such a significant increase in scattering intensity, other factors need to be considered. Related studies indicate that for gold nanorods, the calculation of their shape, aspect ratio, and size effects on absorption and scattering efficiencies show that while the wavelength range for maximum scattering and absorption coincides when the structure size is fixed, the optimal size for maximizing scattering differs from that for maximizing absorption [46]. This implies that if the size or shape of the structure changes, and either scattering or absorption increases while the other decreases, the reflection extinction spectrum, which represents both contributions, may not change significantly. Furthermore, evaluations of the optical properties of various shapes of Ag nanostructures using DDA calculations reveal that for structures with a diameter of 40 nm, the contribution of the scattering coefficient to the extinction coefficient is less than 10% for the disk-shaped structure, whereas for the spherical structures, the contribution is about 30% [47]. This indicates that the proportion of light re-emission from localized surface plasmons is greater in spherical shapes than in disk shapes. Therefore, it is possible that the shape changed from disk-shaped to hemispherical due to annealing, leading to a significant increase in scattering intensity and a slight increase in reflected extinction intensity at the same time due to weakened absorption.

**Figure 5:**
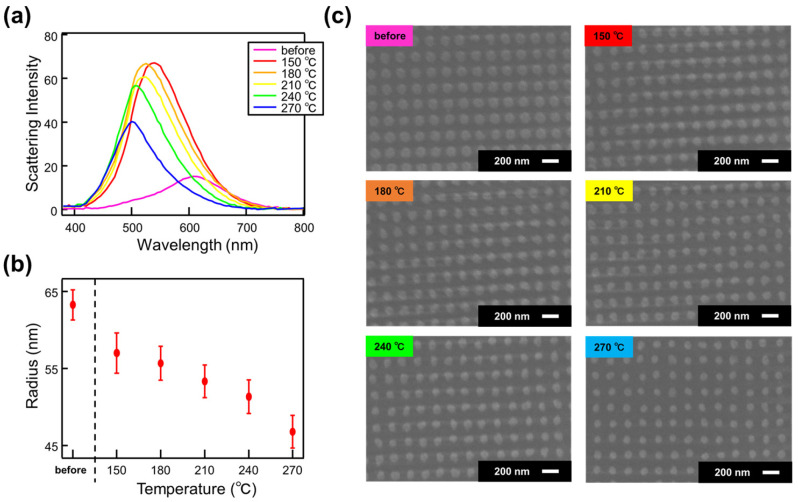
(**a**) Scattering intensity, (**b**) structure radius, and (**c**) SEM image of NDoG structure as a function of annealing temperature.

**Figure 6:**
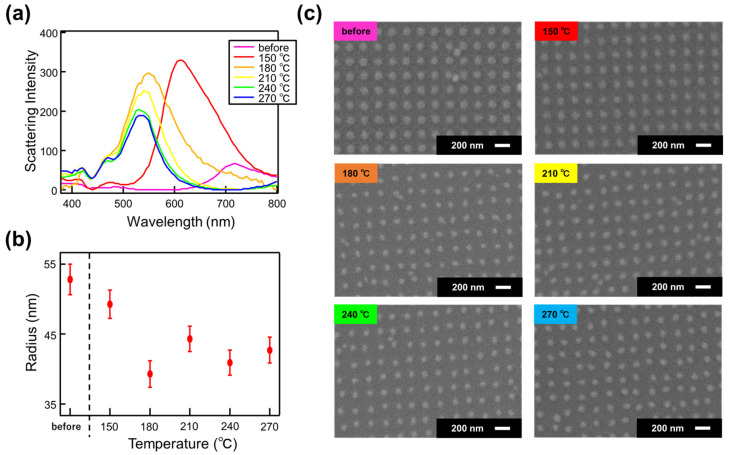
(**a**) Scattering intensity, (**b**) structure radius, and (**c**) SEM image of NDoM structure as a function of annealing temperature.

To further elucidate the changes in optical properties due to annealing, we performed annealing on both the NDoM and NDoG structures at intervals of 30 °C, ranging from 150 °C to 270 °C. We then measured the scattering intensity and observed the surface morphology using SEM. Figure 5 and Figure 6 show the scattering intensity, SEM images, and particle size analysis for the NDoG and NDoM structures, respectively, after annealing at each temperature.

As shown in Figure 5a, the scattering intensity increases upon annealing at 150 °C for the NDoG structure, similar to the NDoM structure. This increase in scattering intensity is attributed to the shape change from a disk to hemispherical form caused by annealing. Additionally, annealing at temperatures above 180 °C results in a shift of the resonance peak to shorter wavelengths and a decrease in scattering intensity. As shown in Figure 5b,c, annealing above 180 °C causes a decrease in structure size, leading to a reduction in the surface area contributing to scattering, which in turn results in the observed shift in the resonance peak and decrease in scattering intensity.

A similar trend is observed for the NDoM structure shown in Figure 6. The increase in scattering intensity after annealing at 150 °C is attributed to the shape change, similar to the NDoG structure. At higher annealing temperatures, the decrease in structure size is believed to cause a deviation from the size that maximizes scattering due to the stronger confinement of the electric field in the 15 nm spacer layer structure. Despite using similar design conditions, the disk structure in the NDoG structure is larger than in the NDoM structure. This difference is likely due to variations in the backscattering coefficient of the electron beam during electron beam lithography, which resulted in more extensive exposure to the glass substrate. Additionally, the slight reduction in disk size observed at 180 °C in Figure 6 is attributed to potential discrepancies in the observation position, focus adjustment, and charge-up effects during SEM observations.

**Figure 7:**
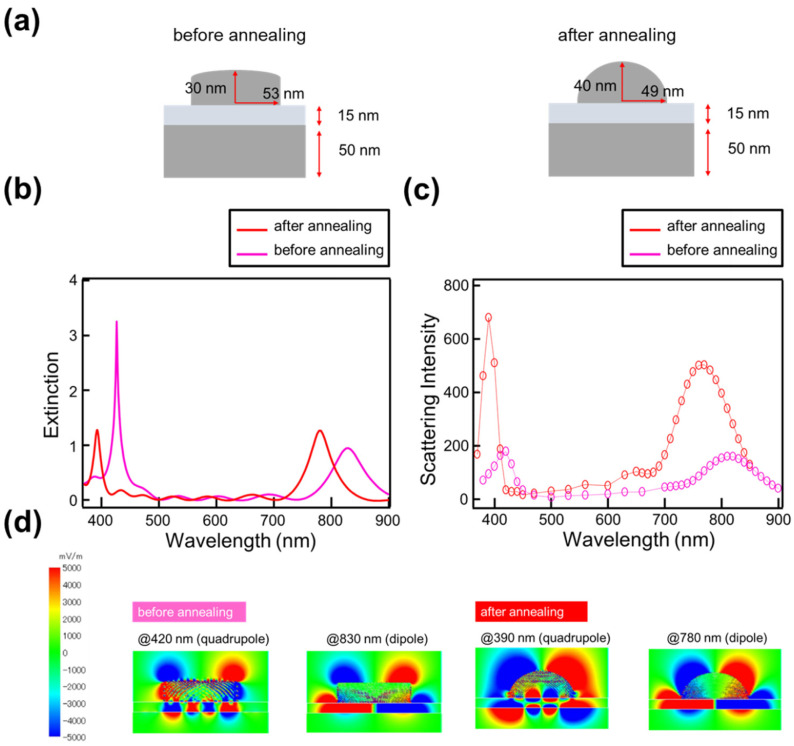
(**a**) Shape changes before and after annealing, (**b**) extinction changes, and (**c**) scattering intensity changes associated with these shape changes. The circles in the figure show the calculated scattering intensity at each wavelength. (**d**) Electric field distribution before and after annealing in the wavelength region where both the short- and long-wavelength peaks are observed.

To investigate the impact of shape changes on scattering intensity, simulations were conducted using the FDTD method considering the transition from disk to spherical shapes, aiming to reproduce the experimental results. Figure 7a illustrates the assumed shape changes. Based on SEM observations, the radius of the nanostructure decreased from approximately 53 nm to 49 nm before and after annealing. For the pre-annealing structure, a disk shape with a radius of 53 nm and a height of 30 nm was used, consistent with the experimental results and design. After annealing at 150 °C, the radius was adjusted to 49 nm, and the structure was assumed to have transformed into an elliptical hemisphere with a height of 40 nm. The results of the extinction and scattering intensity for each shape are shown in Figure 7b,c. The results demonstrate that the slight increase in extinction, the significant enhancement in scattering intensity, and the shift of each peak to shorter wavelengths, as observed in Figure 4, were successfully reproduced. This supports the interpretation that the structure approached a hemispherical shape due to annealing, leading to a substantial increase in scattering intensity. Figure 7d shows the electric field distribution at the wavelengths corresponding to the short- and long-wavelength peaks observed in the structure both before and after annealing. The localization of the electric field is more pronounced in the structure after annealing, which is considered to be one of the key factors contributing to the significant enhancement in scattering intensity.

Since the shape of the nanostructures changes due to annealing, the thickness of the spacer layer that induces the maximum resonance peak also changes. It has been reported that the spacer layer thickness dependence of plasmon resonance for hemispherical nanostructures on top of a mirror and spacer layer exhibits behavior similar to that of disk-shaped structures [36]. As the spacer layer becomes thinner, the resonance peak wavelength shifts to longer wavelengths. Additionally, as the radius of the hemisphere increases, the optimal spacer layer thickness for achieving the strongest plasmon resonance becomes thinner. If the shape changes caused by annealing (i.e., radius and height) can be predicted, it would be possible to optimize the spacer thickness to achieve the maximum resonance peak after annealing.

In addition to changes in shape, the increase in oscillation strength due to annealing may also contribute to the increase in scattering intensity. We previously reported on the annealing effects in NDoG structures using simulations that applied a Drude model adjusted with values from the Lorentz–Drude (LD) model to the dielectric functions of Au and Ag, focusing on a limited spectral range [48]. Assuming that background factors such as electronic transitions remain relatively unchanged, two parameters in particular need to be considered are oscillation strength (OS) and damping frequency (DF). An increase in OS leads to a blueshift in the peak, while an increase in DF broadens the peak while maintaining its wavelength. The improvement in metal quality due to the reduction in grain boundaries from annealing promotes collective electronic oscillations, which may be associated with an increase in OS and a decrease in DF [49,50]. Simulations reproducing the experimental results for the NDoG structure showed an increase in OS. Since an increase in OS also implies an increase in the real part of the dielectric function, it is possible that the annealing contributes to a significant enhancement of scattering intensity. These results suggest that, in addition to the shape of the material becoming more hemispherical due to annealing, the reduction in structural defects and the increase in the number of electrons contributing to collective oscillation of electrons also contributed to the significant increase in the scattering intensity.

The enhancement of scattering intensity achieved through annealing is important for applications such as luminescence enhancement. The approach of using advanced microfabrication technology to create highly controlled, ordered structures, combined with annealing as a post-processing step under optimal conditions, has proven effective in precisely tuning resonance peak wavelengths and significantly increasing scattering intensities. These findings are particularly beneficial for applications such as luminescence enhancement, which depends on the re-emission of light from the LSPR.

## 4. Conclusions

In this study, the optical properties of the NDoM structure were analyzed using FDTD calculations and experimental fabrication through EBL. The calculation results revealed that the NDoM structure exhibited two LSPR peaks in the visible region, and these peaks could be controlled by adjusting the disk diameter and spacer layer thickness of the structure. The fabricated samples showed similar peaks in the visible range as those predicted by the simulations, and it was found that the peak wavelengths could be tuned by changing the structural size through annealing. Additionally, we found that annealing the NDoM structure at 150 °C for 5 min in a nitrogen atmosphere enhanced the scattering intensity by a factor of about five. This enhancement of scattering intensity was attributed to the shape change and crystallinity improvement due to annealing. This enhancement of scattering intensity by post-annealing of structures fabricated on silver mirror substrates using EBL is a novel finding, and it represents an important approach to improve the properties of nanoscale metallic structures. In the future, we plan to further analyze these results in detail by performing similar treatments on structures of different metallic materials and geometries fabricated using EBL, calculating detailed scattering and absorption intensities using discrete dipole approximation (DDA), and analyzing surface conditions using atomic force microscopy (AFM).

## Data Availability

The data presented in this study are available from the corresponding author upon request.
